# Flow Reactor Study of the Soot Precursors of Novel Cycloalkanes as Synthetic Jet Fuel Compounds: Octahydroindene, *p*‑Menthane, and 1,4-Dimethylcyclooctane

**DOI:** 10.1021/acs.energyfuels.5c03795

**Published:** 2025-10-30

**Authors:** Samah Y. Mohamed, Nimal Naser, Zhanhong Xiang, Gina M. Fioroni, Charles S. McEnally, Robert L. McCormick

**Affiliations:** ‡ 53405 National Renewable Energy Laboratory, Golden, Colorado 80401, United States; § Department of Chemical and Environmental Engineering, 5755 Yale University, New Haven, Connecticut 06520, United States

## Abstract

Sustainable aviation fuels (SAFs) or Synthetic aviation turbine fuels (SATFs) derived from nonpetroleum sources are essential for energy security and a strong rural and agricultural economy. Airplanes operating on SAF can have lower particle emissions compared to those of conventional jet fuel, reducing air quality impacts near airports. Processing biobased isoprene or wood and agricultural waste can produce cycloalkane-rich fuels with properties meeting ASTM International’s SATF requirements. The unique structures of these cycloalkanes yield lower soot emissions because of their lack of aromatic rings. We measured the soot formation tendency as yield sooting index (YSI) and used laminar flow reactor experiments to evaluate soot precursors formed for isoprene-derived compounds *p*-menthane and 1,4-dimethylcyclooctane (DMCO), and octahydroindene (OHI)— produced from woody biomass via catalytic fast pyrolysis. The combustion chemistry of the OHI and DMCO has not been previously studied. Experiments were conducted at 10 bar from 800 to 1200 K, equivalence ratios of 1.0 and 3.0, and residence times of 1.0 and 0.6 s, respectively. Experimentally detected species were used to elucidate the mechanisms of soot precursor formation. OHI exhibited the highest YSI (94.5) and formed a high concentration of benzene primarily by direct dehydrogenation of the six-membered ring. *p*-Menthane (YSI 92.0) and DMCO (YSI 85.0) oxidation products included fewer aromatic components but higher benzene precursors, including 1,3-butadiene, propyne, and allene. This suggests that the ring-opening pathway is dominant over the dehydrogenation pathway in the benzene formation for these compounds. This experimental speciation provides insight into the influence of the cycloalkane structure on the sooting tendencies of potential SAF blend components, thereby aiding in fuel design processes.

## Graphical abstract

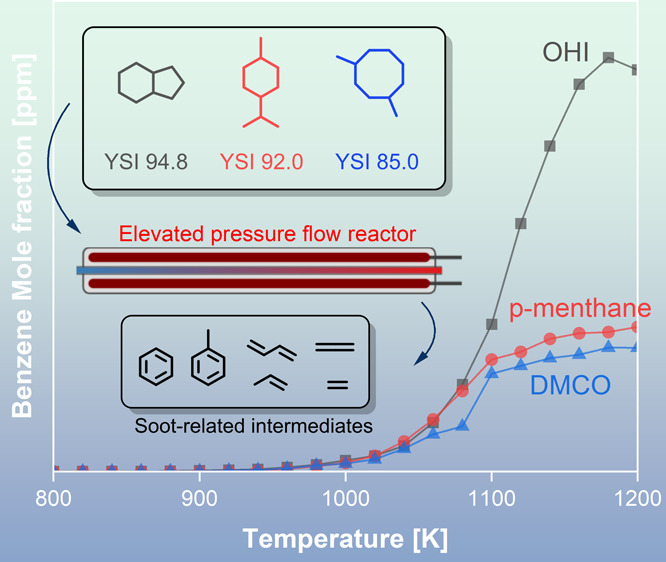

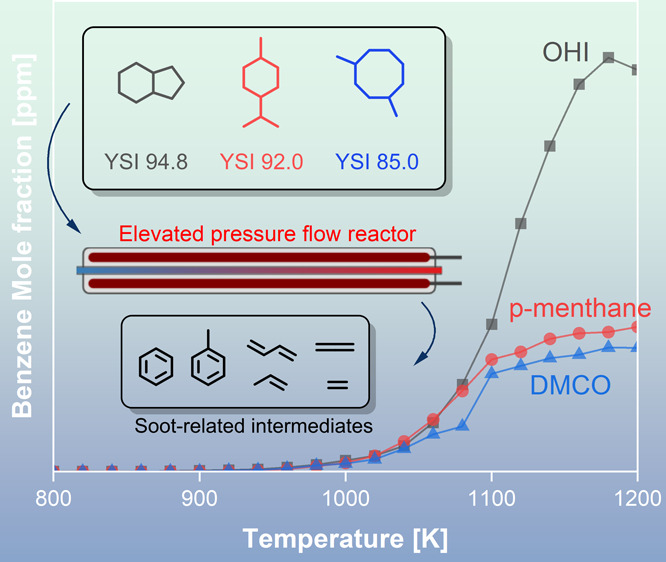

## 1 Introduction

Soot emissions from the aviation sector can negatively impact air quality near airports, with aircraft engines as the dominant source of fine particles.^1–3^ The development of cleaner-burning sustainable aviation fuels (SAFs) or synthetic aviation turbine fuels (SATFs) is a key strategy for mitigating aviation-related emissions while at the same time expanding the feedstocks used to make jet fuel with associated economic benefits to agricultural communities. Currently, approved SAF pathways predominantly yield *n*-alkanes and iso-alkanes, whereas conventional jet fuel contains significant amounts of aromatics and cycloalkanes. To ensure compatibility with the existing infrastructure, SAF must be blended with aromatics and cycloalkanes to replicate the properties and performance of conventional jet fuel. Aromatics in jet fuel are critical for elastomer swelling to maintain engine fuel system integrity. However, reducing or replacing aromatic components can lower soot emissions and improve engine efficiency.^4–6^ Cycloalkanes, such as decalin and monocyclic alkanes, have demonstrated elastomer swelling behavior comparable to Jet A fuel when blended at approximately 30 vol %.^4,7,8^ These promising properties, combined with high specific heat and energy density, make cycloalkanes a potentially viable replacement for aromatics when blended in synthetic paraffinic kerosene (SPK).^4,9^ SPK includes hydrogenated esters and fatty acids (HEFA-SPK), which are the primary commercially available forms of SAF blendstock today.

The development of innovative production routes for sustainable jet fuel blends from biomass resources has led to many pathways dominantly yielding cycloalkane compounds.^10^ For instance, catalytic fast pyrolysis (CFP) of lignocellulosic biomass produces bio-oil that is predominantly hydroxyaromatics, which is subsequently converted via hydrodeoxygenation and hydrogenation to form cycloalkanes. Subsequent distillation yields gasoline, diesel, marine, and SAF fractions. The SAF fraction primarily consists of cycloalkanes (>89 wt %) with minor amounts of alkanes (3–5 wt %), iso-alkanes (2–3 wt %), and aromatics (3–4 wt %). The cycloalkane fraction is composed of alkylcyclohexanes such as propylcyclohexane and 1-methyl-2-propylcyclohexane, along with fused-ring compounds such as decalin and octahydroindene (OHI). Measured properties of this fraction meet blendstock requirements in the ASTM D4054 standard and after blending with jet fuel can meet the requirements of ASTM D7566 for SATF.^11^

Beyond lignocellulosic biomass, cellulose- and hemicellulose-derived terpenes, which are composed of isoprene units, present another promising source of cyclic compounds. Hydrogenation of α-terpinene or limonene in the presence of a catalyst yields *p*-menthane with minor traces (∼2%) of *p*-cymene;^12^ 100% conversion of 1,8-cineole to *p*-menthane was also achieved in a biphasic catalytic process.^13^ Keller et al.^14^ reported that the dehydrogenation, isomerization, and hydrogenation of linalool yielded a mixture of 60% *p*-menthane, 35% 2,6-dimethyloctane, and 4% *p*-cymene, demonstrating promising properties for jet fuel blending. Alcohols, cyclic ethers, and acetate have also been identified as viable precursors for *p*-menthane.^14^ *p*-Menthane showed favorable density, net heat of combustion (NHOC), and viscosity (at −40 °C). A 10 vol % *p*-menthane blend with HEFA-SPK meets viscosity and NHOC specifications; however, at least 32 vol % *p*-menthane is required to achieve ASTM density requirements.^12^

Moreover, isoprene can undergo thermal dimerization to cyclic monoterpenes, followed by catalytic hydrogenation, leading to a high-performance hydrogenated isoprene dimer blendstock consisting of C_10_H_20_ branched cyclohexane isomers, including *p*-menthane and traces of *p*-cymene.^15,16^ Alternatively, catalytic dimerization of isoprene to 1,6-dimethyl-1,5-cyclooctadiene followed by hydrogenation can yield the cyclic compound 1,4-dimethylcyclooctane (DMCO). DMCO’s unique molecular structure results in desirable properties, making it a promising jet fuel and SPK blending component. Its cyclic structure increases the density and NHOC, while branching contributes to lower viscosity and a reduced freezing point. DMCO maintains low viscosity at −20 °C and −40 °C, enabling efficient atomization and easy relight at altitude.^4^ A 30 vol % DMCO blend into HEFA-SPK meets all ASTM specifications for density and viscosity, except for electrical conductivity, which can be corrected with additives.^15^

The advantageous properties of cycloalkanes, including a low melting point and high specific energy and energy density, highlight their potential as SAF or SAF blend components. However, a comprehensive understanding of their combustion properties and the effect of molecular conformation and branching does not exist. Notably, cycloalkanes exhibit a higher yield sooting index (YSI) than *n*-alkanes or iso-alkanes,^17^ as shown in Figure 1, with the higher-energy-density bicycloalkanes exhibiting the highest YSI. Nonetheless, cycloalkanes still exhibit significantly lower YSI than aromatics (monoalkyl benzene). The structural complexity of cycloalkanes goes well beyond those shown in Figure 1, and it has been observed that YSI increases with the number of alkyl groups attached to a cyclohexane ring.^17,18^

**Figure 1:**
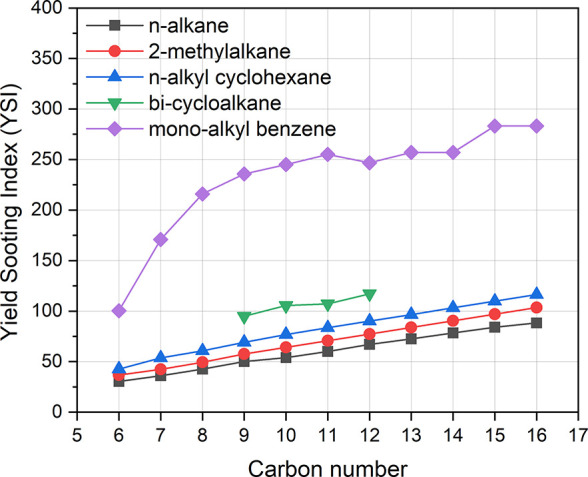
YSI versus carbon number for *n*-alkanes, 2-methylalkanes, *n*-alkyl cyclohexane, bicycloalkanes (OHI, *cis*-decalin, 2-methyl decalin, and bicyclohexane), and monoalkyl benzene. The data are a mixture of measured and predicted values taken from the National Renewable Energy Laboratory’s YSI prediction tool.^19^

Depending on the oxidation conditions, cycloalkanes, specifically cyclohexane, can either undergo ring opening to straight-chain intermediates that further decompose into smaller species or proceed via the dehydrogenation pathway, leading to benzene formation. The former pathway is prevalent in low-temperature oxidation and stoichiometric premixed flames, whereas the latter dominates in high-temperature, fuel-rich, nonpremixed flames.^20,21^ Benzene and higher aromatics (2–5 rings) are commonly considered key soot precursors.^22^ The formation of these precursors is strongly influenced by the molecular structure of the fuel components and has been found to corelate with the fuel’s index of hydrogen deficiency (IHD), which accounts for the cyclic structure and degree of unsaturation in a hydrocarbon.^23^ Fuels with higher IHD produce higher aromatics concentration in soot precursor experiments,^20^ supporting the trend in soot precursor formation of aromatics > cycloalkanes > iso-alkanes > *n*-alkanes. This highlights the benefit of reducing the aromatic content in jet fuels to lower emissions and mitigate soot formation.

Xu et al.^24^ theoretically and experimentally investigated the effect of ring size and branching in the sooting tendency of various cycloalkanes, specifically cyclopentane (cC_5_H_10_), cyclohexane (cC_6_H_12_), and methylcyclohexane (cC_7_H_14_). Their laser-induced incandescence experiments showed a trend of sooting cC_5_H_10_ > cC_7_H_14_ > cC_6_H_12_. This trend contrasts with the YSI trend of cC_5_H_10_ (YSI 39.4) < cC_6_H_12_ (YSI 42.7) < cC_7_H_14_ (YSI 53.6).^19^ While they acknowledged the difficulty in pinpointing the source of the discrepancy, they suggested that the chemical interaction between the methane and the fuel during the YSI experiment could be a contributing factor. Furthermore, to understand the underlying mechanism, Xu et al.^24^ performed closed homogeneous reactor simulation demonstrating that cC_5_H_10_ produces relatively more benzene and C_3_H_3_ (an important benzene precursor) compared to cC_6_H_12_. Both cC_5_H_10_ and cC_6_H_12_ decompose via ring opening to form an alkene, followed by C_3_H_5_-a formation. However, cC_5_H_10_ radicals (cC_5_H_9_) tend to decompose into odd-carbon intermediates that eventually form C_3_H_3_, whereas cC_6_H_12_ radicals (cC_6_H_11_) decompose into C_4_ and C_2_ species, which contribute less to benzene formation compared to C_3_H_3_. These findings align with those of Kathrotia et al.,^20^ who identified C_4_H_6_, C_2_H_4_, and benzene as major intermediates in cyclohexane, confirming the significance of both ring opening and the dehydrogenation pathway for soot precursor formation. Their study also examined the sooting mechanism of different hydrocarbons including decalin (bicycloalkane), in which the decalyl radical forms cyclohexene, toluene, cyclopentadiene, styrene, and ethylbenzene, all of which contribute to benzene formation.^20^ Additionally, decalin was found to be a major contributor to the two-ring aromatic species, such as indene and naphthalene, through partial decomposition and dehydrogenation reactions.^25^

The sooting behavior of cycloalkanes has also been analyzed in the context of soot precursor formation from 26 different jet fuels studied in a flow reactor.^22^ The formed intermediates, particularly soot precursors, were associated with the fuel’s components. Acetylene, butadiene (C_4_H_6_), and benzene formation showed a strong correlation with the cycloalkane concentration in the fuel. Acetylene primarily formed via the ring opening of cyclic components, leading to the cyclopentadienyl radical (C_5_H_5_), butynyl radicals (C_4_H_5_), and eventually acetylene. The dehydrogenation of cyC_6_H_10_ (cyclohexene) has been proposed as a key to benzene formation from cyclohexane.^22^

The sooting tendency and YSI are also influenced by the functional groups present in the cyclic compounds. For instance, the position of a double bond in methylcyclohexene isomers resulted in a variation of up to 24 YSI points, with 3-methyl-1-cyclohexene exhibiting the highest YSI of 82.0 compared to 1- and 4-methyl-1-cyclohexene.^26^ Kim et al.^27^ investigated the primary mechanism of soot precursor formation in the methylcyclohexene isomers using density functional theory combined with a flow reactor experiment at stoichiometric and ambient conditions. The findings revealed that the retro-Diels–Alder reaction is a major consumption pathway for the predominant radicals formed in 1- and 4-methyl-1-cyclohexene. However, in 3-methyl-1-cyclohexene, the radical that would typically undergo the retro-Diels–Alder reaction instead isomerizes to a more stable radical, which subsequently promotes toluene formation, leading to a higher sooting tendency. A similar approach, combining flow reactor experiment and density functional theory calculation, was employed to investigate the structural effect on soot precursor formation pathways for phenylethanol^28^ and ether isomers.^29^

In this study, we used a flow reactor to experimentally investigate the sooting tendency and soot precursor formation of three bioderived cycloalkanes, each with significant potential as SAF or as a SAF blending component. The selected cycloalkanes are shown in Figure 2 and represent diverse structures including (1) OHI, a major fused-ring component of CFP-derived SAF fuel that has not been studied previously; (2) *p*-menthane, a branched cyclohexane that demonstrates the predominant class of cycloalkanes in both jet fuels and CFP-derived SAF; and (3) DMCO, a strained 8-membered ring cycloalkane, as there are limited data available on the sooting behavior of large-ring cycloalkanes.

**Figure 2:**
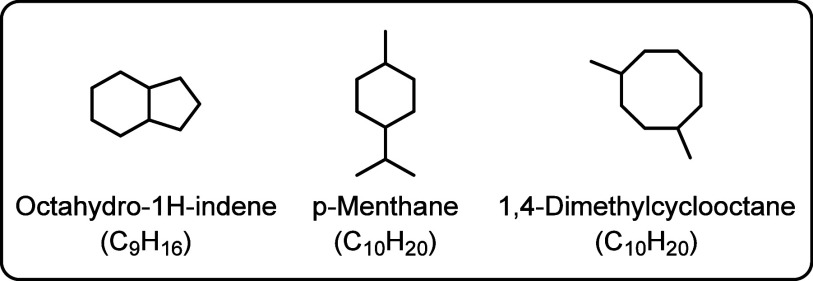
Considered cycloalkane structures.

DMCO and *p*-menthane are derived from isoprene. Several studies have reported on the production pathways and properties of isoprene-derived molecules, but kinetic studies on their combustion behavior and sooting tendency remain limited. Oßwald et al.^30^ experimentally investigated the combustion kinetics of terpene-based compounds, including farnesane, *p*-menthane, and *p*-cymene, in a high-temperature flow reactor experiment coupled with molecular beam mass spectrometry. The experiments were performed at equivalence ratios of 0.5, 1.0, and 1.5 over a temperature range of 800–1050 K. Under these conditions, benzene formation from *p*-menthane was primarily attributed to propargyl combination, as the concentration of C_4_H_5_ species was low. Notably, higher levels of polycyclic aromatic hydrocarbons were detected only for the aromatic compound *p*-cymene. Gong et al. further investigated the pyrolysis of *p*-menthane at both atmospheric and elevated pressures in a microreactor experiment analyzed by an online gas chromatography–mass spectrometry/flame ionization detection (GC–MS/FID) system.^31,32^ They also developed lumped^31^ and detailed RMG-based^32^ pyrolysis kinetic models, both of which agreed well with the experimental results. Their findings indicated that H-abstraction and side-chain scission (isopropyl or methyl group) were the primary consumption pathways for *p*-menthane, with unimolecular decomposition being more favorable at low pressures. Additionally, molecular dynamics simulations of *p*-menthane pyrolysis at 2600 K revealed that unimolecular decomposition to release the isopropyl side group was the dominant pathway.^33^ Production of OHI as a component of the CFP-derived jet has been described,^11^ but only limited fuel property data have been previously reported, and we are not aware of previous combustion kinetics studies.

## 2 Methods

### 2.1 Fuels

The properties of the studied cycloalkanes, along with properties of *n*-decane and decalin for comparison, are summarized in Table 1. These pure compounds would be limited to low blending levels in jet fuel because of their impact on the distillation curve. Nevertheless, it is informative to compare their properties to the fast track property guidelines in ASTM D4054 and the requirements for aviation fuel containing synthesized hydrocarbons in ASTM D7566. Density is required to be between 0.730 and 0.800 g/mL for synthetic blendstocks. Generally, the cycloalkanes have a density above this range. All meet the maximum viscosity limit at −20 °C, but the OHI exceeds the maximum limit at −40 °C (viscosity of *n*-decane and decalin could not be measured at −40 °C, as this is well below their freezing points). NHOC for all finished jet fuels is required to be at least 42.8 MJ/kg, which is met by all the pure compounds except OHI. In terms of energy density, typical Jet A is around 35 MJ/L, with all the cycloalkanes at that level or higher and with both bicycloalkanes at about 38 MJ/L—a significant improvement. The cetane number, as either derived cetane or indicated cetane, is required to be 35 or greater for alternative jet fuel blending components for adequate resistance to lean blowout. Of the cycloalkanes, only decalin meets this requirement. YSI is not a requirement of any jet fuel or SAF standard, but it is most relevant for this study. The derived smoke points are calculated from the YSI values using a linear fit^34,35^ that has been shown to agree well with experimental smoke point measurements.^36^ The cycloalkanes meet the smoke point requirements. As expected, the cycloalkanes have significantly higher YSI and lower derived smoke points compared to *n*-decane.

**Table 1:** Properties of OHI, *p*-Menthane, and DMCO Compared to the Limits for Synthetic Blendstocks in D7566 and D4054 (*n*-Decane and Decalin Included for Comparison)

property	property limits (D7566/D4054)	*n*-decane^a^	DMCO^b^	*p*-menthane^c^	OHI^a^	decalin (*cis*/*trans* avg.)^a^
density at 15 °C(g/mL)	>0.730	0.755	0.827	0.801	0.887	0.881
η(−20 °C) [mm^2^·s^–1^]	<8.0	2.66	4.17	2.98	7.47	8.68
η(−40 °C) [mm^2^·s^–1^]	<12.0		7.95	5.15	15.76	
NHOC (MJ·kg^–1^)	>42.8	44.6	43.82	43.20	42.47	43
NHOC (MJ·L^–1^)		32.74	36.22	34.72	37.67	38.1
flash point (°C)	>38		50	44.6	42.8	58
freezing point (°C)	<−40	–27	<−78	–55	–56	–29
boiling point (°C)		174	172	168	164	191
surface tension at 20 °C (mN/m)		23.8^d^	25.9^a^	24.5^a^	30.7	31.0^d^
YSI		54.0^e^	85.0	92.0	94.8	105.5^e^
derived smoke point (mm)^a^	>25 or 18^f^	100	40	35	28	27
derived cetane no.	35 min/60 max	66^g^	18	29		35.6^g^
indicated cetane no.	35 min/60 max	71	14		24	39.5

a Measured or calculated for the present work.

b DMCO properties from refs 4,15 , and^34^.

c *p*-Menthane properties from refs 12,16 , and^37^.

d Surface tensions of *n*-decane and decalin from ref 38.

e *n*-Decane and decalin YSIs from ref 19.

f If fuel contains a maximum of 3 vol % naphthalenes.

g *n*-Decane and decalin derived cetane numbers from ref 39.

### 2.2 Flow Reactor Experiments

The speciation profiles during the oxidation of the three cycloalkanes were obtained with a pressurized laminar flow reactor. This reactor consists of a steel tube with an oxygen-resistant SilcoTek coating. The reactor has an internal diameter of 1.3 cm and length of 71.1 cm and is seated in a furnace that can be heated to 1200 K. The reactor was operated at a pressure of 10 bar. Fuel is injected into the packed bed column swept by preheated helium (diluent) and then into the reactor. The temperature of the mixing column is maintained at 470 K for proper fuel vaporization and mixing. Oxygen is separately introduced into the inlet of the reactor. Based on the required reactor conditions of the equivalence ratio, residence time, and inlet fuel mole fraction, the fuel, oxidizer, and diluent flow rates are adjusted with mass flow controllers. In this study, the speciation profiles under soot-precursor-forming conditions were obtained at equivalence ratios of 1 and 3 and an inlet fuel mole fraction of 250 ppm. A detailed schematic of the experimental setup and flow rates of fuel, oxidizer, and diluent are provided in the Supporting Information (SI). A detailed description of the National Renewable Energy Laboratory’s laminar flow reactor is available elsewhere.^40–42^

In the laminar flow reactor under fuel-rich conditions, if the residence time is too long, high-carbon-number soot or polycyclic aromatic hydrocarbons can be formed. Many of these polycyclic aromatic hydrocarbons have high melting points and can easily condense in the sampling lines and GC sampling loops, resulting in experimental difficulties, carbon balance issues, and higher experimental uncertainties. To avoid this, the residence time had to be kept short, and 0.6 s was selected for all of the cycloalkanes in this study.

The effluent from the reactor was analyzed with two separate GC systems. The temperatures of the effluent sampling line and GC sampling loops were maintained at 420 and 520 K, respectively, to prevent condensation of products. The heavier species (≥C_5_) were identified and quantified on GC1, which uses a 60 m × 320 μm × 1 μm capillary column, and the column effluent splits to two detectors in parallel. The detectors are (1) an FID coupled to a Polyarc methanizer and (2) a MS. The The Polyarc methanizer is a catalytic microreactor that converts all the effluent species to methane before detection in the FID. Conversion to methane allows for increased sensitivity and accurate quantification of the oxidation products. *n*-Heptane was used to calibrate the methanizer, and the species were identified with an MS spectrogram using the National Institute of Standards and Technology database.^43^ The identified species were quantified with calculated response factors from the FID against the response factor of *n*-heptane calibration runs using the effective carbon number method.^44^ Lighter species (<C_5_) were quantified on GC2 that has an FID and two thermal conductivity detectors. The thermal conductivity detectors were used to quantify CO and CO_2_. The FID of GC2 was calibrated with standard gases with known concentrations (i.e., alkane, alkene, and alkyne standard). The thermal conductivity detectors were calibrated with CO and CO_2_ standards containing 500 ppm each.

Duplicate runs for the entire temperature range were not performed, but triplicate runs for a select data point for each fuel were performed to estimate the standard deviation (SD) of the measurement for the respective fuel. This SD is then applied to all data points of that fuel. In addition to SD, uncertainty analysis was also performed on the species quantified to account for the various experimental uncertainties using linear error propagation theory. Uncertainties can arise due to fluctuations in diluent, oxidizer, fuel flow rates, pressure, and temperature. The uncertainty values chosen for different parameters are given in Table S-4. The uncertainty values in addition to the SD value described above are propagated to obtain the SD of the experimentally quantified species profiles. The average uncertainty in the quantified species is 15%. Greater than 90% of the carbon balance was obtained on an average basis for all the data points presented in this study. Individual carbon balance for the experimental data is available in the SI.

The temperature profiles of the flow reactor for select conditions are available in the SI. Due to measurement constraints at elevated pressures, temperatures only up to half of the reactor could be measured (i.e., the heating and uniform region of the reactor). The temperature profile of the cooling region was assumed to be symmetrical as the heating zone. This assumption may not be accurate, but from in-house simulation studies for related work, it was observed that the cooling region temperature profile has very minor effect on the kinetics. The species have already encountered the maximum temperature in the uniform region and any temperature profile in the cooling region would not alter the chemistry significantly. The heating region profile and uniform temperature region have the most important effect on the chemistries. Liang et al.^45^ investigated the effect of the cooling region temperature profile on the oxidation of *n*-decane and observed that neglecting the cooling region temperature profile influenced the species profiles at some temperature conditions and no effect on other temperatures. It is important that a cooling region temperature profile be included in simulation purposes; however, the profile of the temperature in the cooling region is relatively less influential than that in the heating region where the chemistries build up. As temperature profiles are affected by the set reactor temperature and only limited temperature profile measurements were performed, for any set reactor temperature, the estimated temperature profile was obtained using models provided in the SI.

### 2.3 Yield Sooting Index

Sooting tendencies were measured using a yield-based approach we developed previously.^35^ The specific procedure used in this study is described elsewhere.^34^ It consists of three steps: (1) we sequentially doped 1000 μmol/mol (1000 ppm) of *n*-heptane (H), toluene (T), and each test fuel (TF) into the fuel stream of a base methane/air flame; (2) we measured the maximum soot concentration in each flame with line-of-sight spectral radiance (*L*); and (3) we rescaled the results into a yield sooting index (YSI) defined as

$${\mathrm{YSI}}_{\mathrm{TF}}={\left({\mathrm{YSI}}_{T}-{\mathrm{YSI}}_{H}\right)}^{*}\frac{L_{\mathrm{TF}}-L_{H}}{L_{T}-L_{H}}+{\mathrm{YSI}}_{H}$$ 1

This rescaling eliminates sources of systematic uncertainty, such as the optical properties of the soot. Furthermore, it allows the new results to be quantitatively compared to a database that contains measured YSIs for hundreds of organic compounds.^46^ The parameters YSI_T_ and YSI_H_ are constants that define the YSI scale; their values —170.9 and 36.0— were taken from the database so that the newly measured YSIs would be on the same scale for a direct comparison. The dopants are added at a small concentration to eliminate indirect effects, such as changes in the flame temperature or residence time.

Figure S5 shows a schematic diagram of the apparatus and describes the experiments in detail. Figure S6 gives details of the burner. The liquid test fuels were injected into the gas-phase CH_4_ fuel mixture with a syringe pump. Table S5 lists the liquid-phase flow rates corresponding to 1000 μmol/mol in the gas phase for each test fuel and the property values that were used to calculate them. The fuel lines were heated to 100 °C, and the burner was heated to 170 °C. Each test fuel was injected for 600 s, and *L* was averaged from 300 to 600 s. Figure S7 shows that the initial 300 s is adequate for all the test fuels to equilibrate with the walls of the fuel line and burner. Figure S8 experimentally confirms that the test fuels did not condense in the fuel delivery system.

## 3 Results and Discussion

The products and intermediates detected during the oxidation of cycloalkanes under sooting conditions (equivalence ratio of 3) are analyzed in the following sections. The stoichiometric data (equivalence ratio of 1) are not discussed in the following sections but are provided in the SI. To understand the formation mechanism of reaction intermediates, we outline and discuss their production based on plausible high-temperature reaction pathways. The reaction of cycloalkanes can proceed via H-abstractions or unimolecular decomposition, which includes both ring-opening reactions and C–C bond scission either between the ring and the side chain or within the side chain. In this study, we consider only the H-abstraction and the C–C bond scission between the ring and the side chain as initial reactions. C–C bond scissions within the side chain or ring-opening reactions generally have lower rate constants, making H-abstractions the predominant first reaction step.^32^ The flux diagrams presented focus on high-temperature reaction pathways, including subsequent C–C and C–H β-scissions and isomerizations, as our primary objective is to investigate soot precursor formation.

### 3.1 OHI (YSI 94.8)

The oxidation products of the OHI at 10 bar and an equivalence ratio of 3, along with the proposed consumption pathways, are shown in Figures 3 and 4, respectively. At 800 K, 10% conversion of OHI was observed, with notable consumption and mild change in the conversion slope beyond 1025 K, where most intermediate concentrations peak. Formaldehyde (CH_2_O) was the only detected oxygenate, peaking at high temperatures (∼1150 K), suggesting its formation primarily via methyl radical (CH_3_) oxidation. As formaldehyde decreases, methane (CH_4_) production increases, indicating a shift in methyl radical consumption toward abstraction reactions forming methane instead of formaldehyde. Among the major detected products, ethene (C_2_H_4_) exhibited the highest yield (∼200 ppm at 1150 K), consistent with the multiple reaction pathways leading to its formation in Figure 4. Other detected intermediate and soot precursors include propene (C_3_H_6_), propyne (C_3_H_4_-p), and 1,3-butadiene (C_4_H_6_), with the latter being a major product evident by the OHI decomposition fluxes. The allyl radicals (C_3_H_5_) undergo H-abstraction reactions, forming propene or isomerizing to form 1-propenyl radicals that eventually form propyne. Additional benzene precursors, such as allene (C_3_H_4_-a) and acetylene (C_2_H_2_), were also detected at lower concentrations.

**Figure 3:**
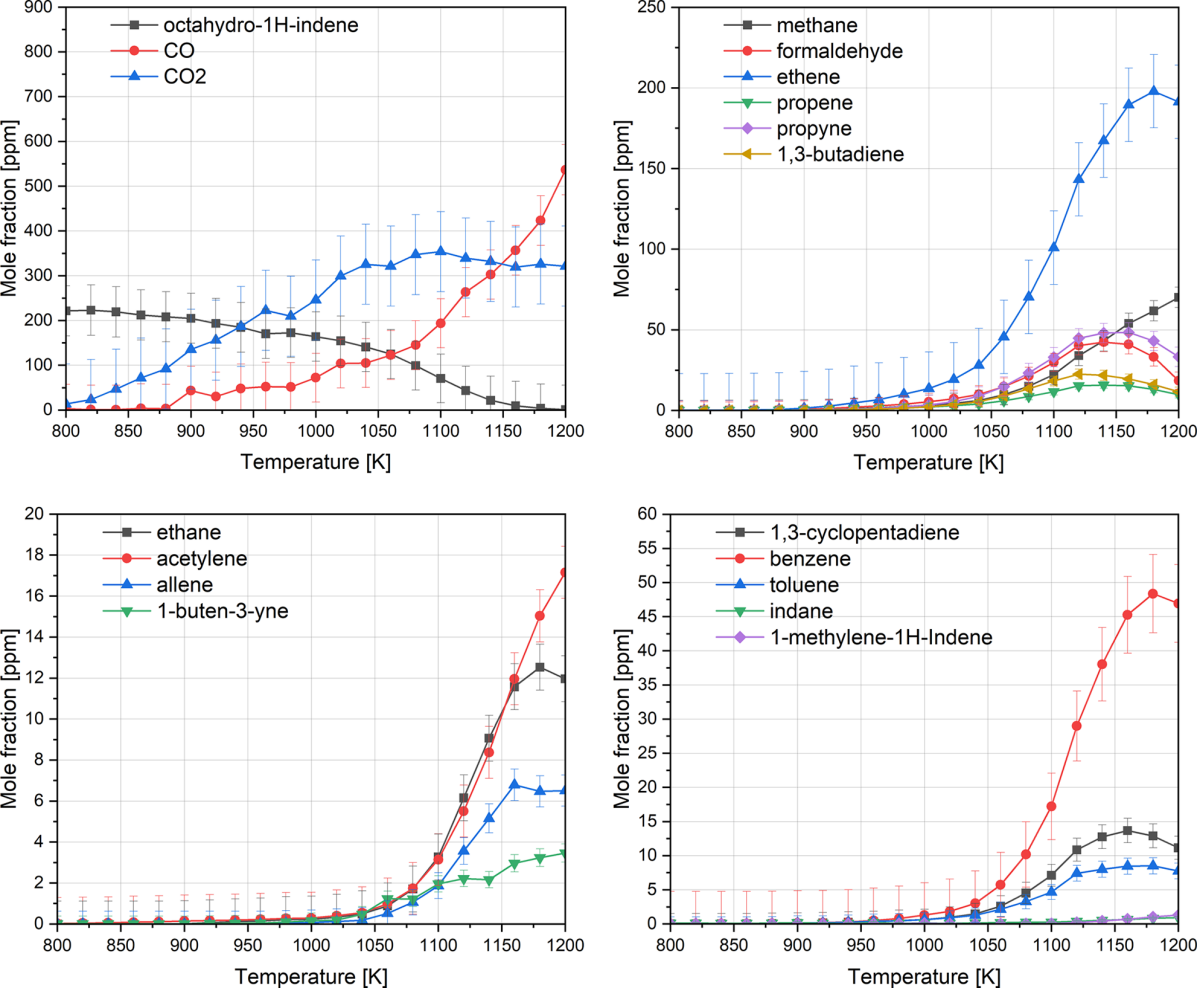
Speciation profiles of OHI oxidation in the flow reactor at 10 bar and phi = 3.0.

**Figure 4:**
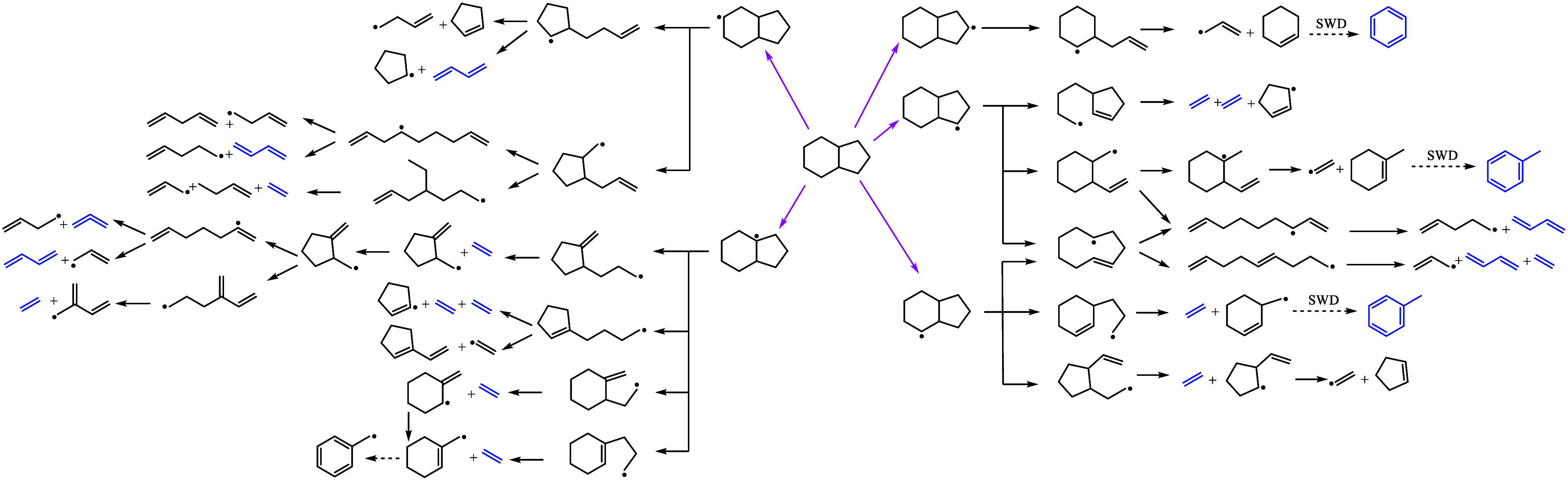
Schematic diagram of the reaction pathways of OHI consumption. Magenta arrows indicate H-abstraction pathways. Blue species were detected experimentally. SWD means the stepwise dehydrogenation pathway.

Aromatic intermediates detected from the OHI oxidation include benzene, toluene, and traces of indane and 1-methylene-1H-indene. Aromatics can be formed by the sequential dehydrogenation of cyclic intermediates or via the recombination of olefins produced from the β-scission of fuel radicals and intermediates.^47^ However, the relatively high benzene concentration (∼50 ppm) suggests that the stepwise dehydrogenation^48^ after 5-membered ring opening plays a key role for OHI. Very similar species observations were made for soot precursor formation from *trans*-decalin,^49^ suggesting that it also forms an aromatic ring by dehydrogenation after opening of the first ring. Moreover, cyclopentene, cyclopentyl, or cyclopentenyl radicals are abundantly produced from 6-membered ring-opening pathways as shown in Figure 4, leading to the formation of the detected 1,3-cyclopentadiene,^50^ which can subsequently yield naphthalene by recombination.^24,51^ These C5-cyclic intermediates can also lead to propargyl radicals and significantly contribute to benzene formation.^52^ A slightly lower benzene concentration was detected under stoichiometric conditions (shown in the SI); however, more cyclic compounds were observed. Overall, the OHI exhibited a similar behavior under stoichiometric conditions with more fuel conversion (50% at 800 K), as indicated by the fuel profile and higher CO and CO_2_ concentrations (given in the SI). Additionally, relatively earlier peaks for intermediates were observed as expected for stoichiometric conditions.^53^

### 3.2 *p*-Menthane (YSI: 92.0)

The detected oxidation products of *p*-menthane at 10 bar and an equivalence ratio of 3 are shown in Figure 5. They primarily consist of C_1_–C_5_ alkanes and alkenes, formaldehyde, aromatics, and trace amounts of cycloalkanes. The possible high-temperature reaction pathways are outlined in Figure 6. Only H-abstraction and unimolecular decomposition via C–C bond cleavage between the ring and side chain were considered as initiation reactions. The detected intermediates were comparable to those reported for *p*-menthane pyrolysis.^32^ *p*-Menthane was gradually consumed up to ∼950 K, beyond which a pronounced change in the consumption slope was observed, coinciding with the beginning of intermediate formation, as shown in Figure 5. The major radicals formed in *p*-menthane decomposition include iso-propyl (i-C_3_H_7_) and methyl radical, generated via unimolecular decomposition of *p*-menthane or the β-scission of the 1-isopropyl-4-methyl-2-cyclohexyl and 1-isopropyl-4-methyl-3-cyclohexyl radicals (Figure 6) due to the low barrier height for side-chain scissions.^54^ The iso-propyl radical undergoes C–H β-scission, forming propene, which peaks at ∼1100 K. Propene can subsequently react with a hydrogen radical to form an *n*-propyl radical (*n*-C_3_H_7_), which then decomposes thermally to ethene and methyl radical.^55,56^ The methyl radical can either oxidize to form formaldehyde or react via H-abstraction to become methane. The inverse behavior of methane and formaldehyde —i.e., decreasing concentration of formaldehyde with increasing concentration of methane—suggests that methyl oxidation shifts from formaldehyde to methane formation as temperature increases. Similarly, both methane and ethene concentrations increase as propene is depleted. Additional detected intermediates include C_4_ and C_5_ alkenes and dienes, shown in Figure 5, produced via various ring-opening pathways, followed by C–C scissions. Among these, 1,3-butadiene was the most abundant, followed by isoprene, 2-butene, and allene. Another major soot precursor, acetylene, exhibited high concentrations at high temperatures. Benzene and traces of toluene were also detected, likely formed by the combination of benzene precursors or the dehydrogenation of cyclic intermediates.

**Figure 5:**
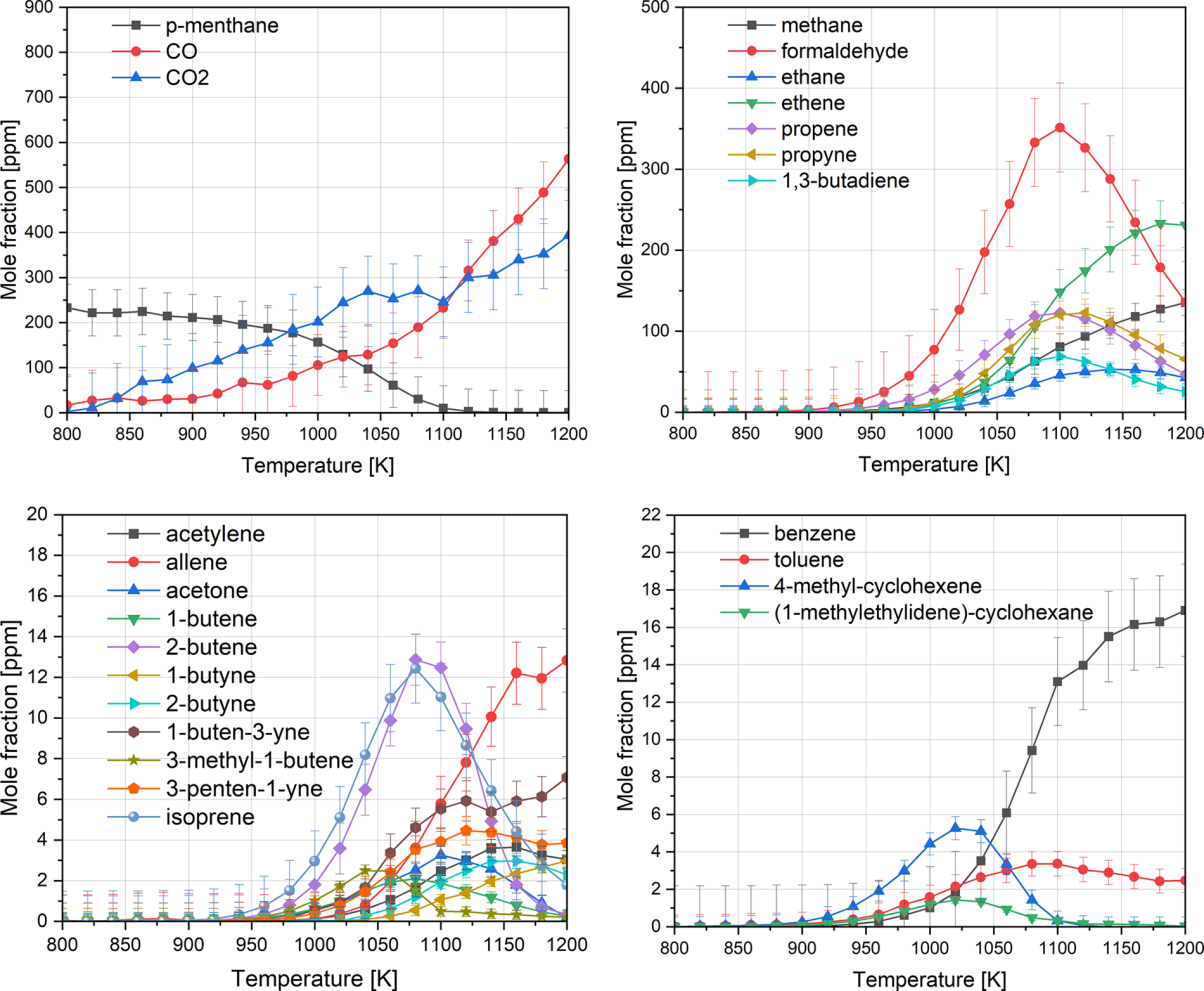
Speciation profiles of *p*-menthane oxidation in the flow reactor at 10 bar and phi = 3.0.

**Figure 6:**
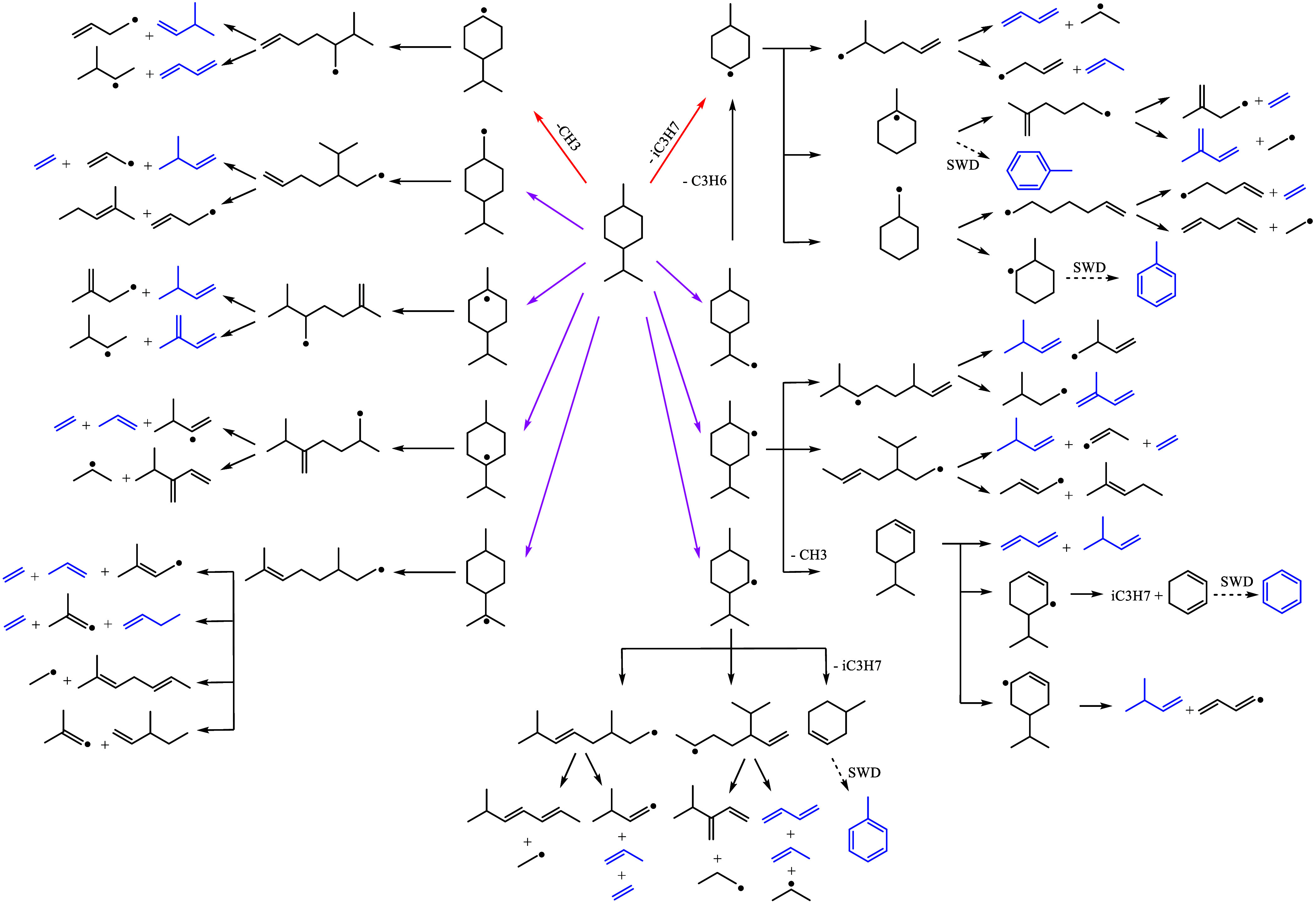
Schematic diagram of the reaction pathways of *p*-menthane consumption. Red arrows indicate unimolecular decomposition pathways, and magenta arrows indicate H-abstraction pathways. Blue species were detected experimentally. SWD means the stepwise dehydrogenation pathway.

Cycloalkanes such as 4-methyl-1-cyclohexene were among the earliest detected intermediates (∼900 K), formed either via *p*-menthane unimolecular decomposition to the 4-methyl-1-cyclohexyl radical or through the β-scission of the 1-isopropyl-4-methyl-2-cyclohexyl radical. This intermediate is a major contributor to aromatics.^31^ Another detected intermediate, (1-methylethylidene)-cyclohexane, likely results from isomerization and C–H scission of the 1-isopropyl-4-cyclohexyl radical, a primary unimolecular decomposition product.

### 3.3 DMCO (YSI 85.0)

The detected oxidation products of DMCO at 10 bar are shown in Figure 7, with the proposed reaction pathways illustrated in Figure 8. The detected *n*-alkenes and iso-alkanes, particularly dienes that peak between 1050 and 1100 K, indicate that DMCO was primarily consumed by ring-opening reactions.^31^ Only minor concentrations of cyclic compounds and aromatics were detected, with benzene reaching a maximum concentration of ∼15 ppm at 1180 K. At 800 K, 12% of DMCO was consumed, yet no oxidation intermediates were observed, suggesting minimal low-temperature chemistry under these fuel-rich conditions. The predominant intermediates included ethene, formaldehyde, propene, propyne, and 1,3-butadiene, possibly originating from β-scission of ring-opening intermediates, as shown in the flux analysis (Figure 8). Methane was produced in significant amounts at higher temperatures, peaking as formaldehyde decreases, as previously indicated. Additionally, both C_4_ and C_5_ alkenes and iso-alkanes resulting from ring opening such as 2,7-dimethyl-octane and 3-methyl-nonane were detected at low concentrations, suggesting rapid consumption into smaller species. Among cyclic compounds and aromatics, benzene was the major intermediate, while other soot precursors such as acetylene and toluene were detected in trace amounts (<2 ppm). Even fewer aromatic and cyclic compounds were detected under stoichiometric conditions (shown in the SI), with benzene being the only identified aromatic reaching a maximum yield of just 5 ppm.

**Figure 7:**
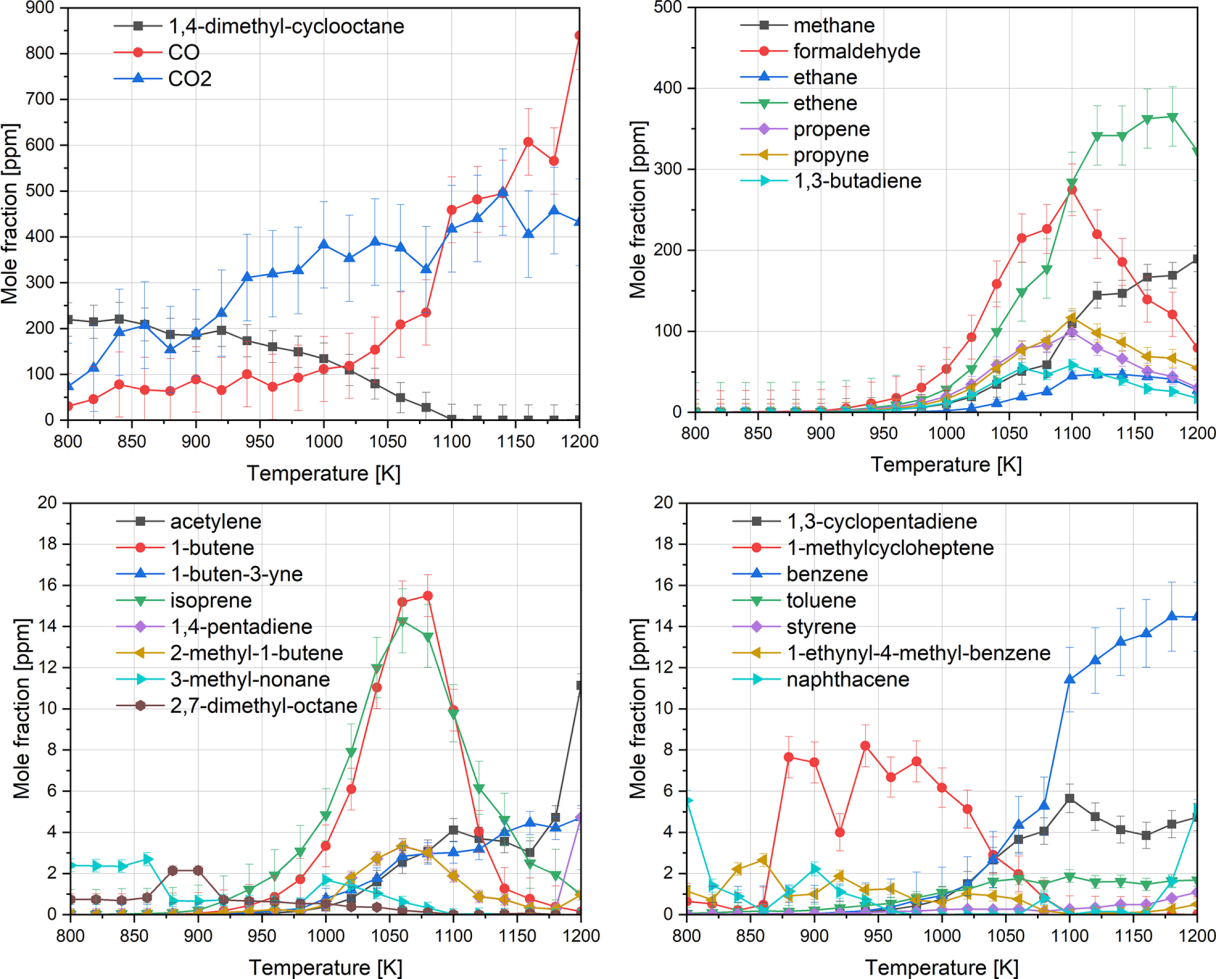
Speciation profiles of DMCO oxidation in the flow reactor at 10 bar and phi = 3.0.

**Figure 8:**
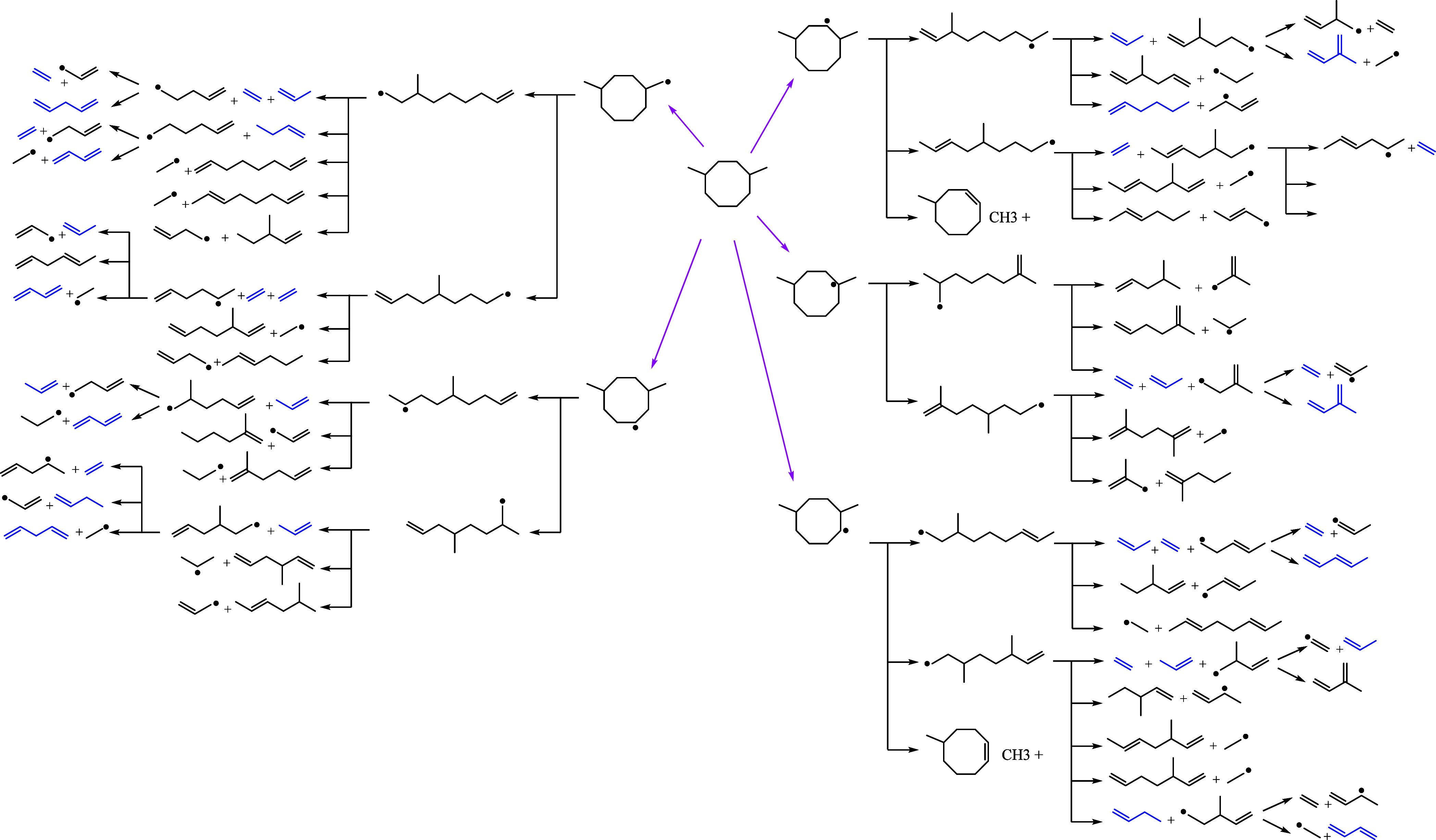
Schematic diagram of the reaction pathways of DMCO consumption. Magenta arrows indicate H-abstraction pathways. Blue species were detected experimentally.

### 3.4 Soot Precursors of OHI, *p*-Menthane, and DMCO

The measured YSI of the three components followed the trend of DMCO (YSI 85.0) < *p*-menthane (YSI 92.0) < OHI (YSI 94.8). Although the small differences in YSI are challenging to explain, the chemical pathways leading to the formation of soot precursors may provide insight as differences are observed. We compared the detected concentrations of acetylene (C_2_H_2_), 1,3-butadiene (C_4_H_6_), propyne (C_3_H_4_-p), and allene (C_3_H_4_-a) in the oxidation products. The last two C_3_H_4_ isomers significantly contribute to the formation of propargyl (C_3_H_3_) radicals, which are key precursors to benzene. Benzene formation can also occur through reactions between C_4_ species (C_4_H_5_) and C_2_ compounds or via the recombination of C_3_ species.^30,57^ Additionally, benzene and toluene profiles and their contribution to YSIs were analyzed, as these species can be directly formed from the parent fuel and contribute to soot formation, depending on the experimental conditions.^20,21^ These concentrations were compared for the species investigated in this work, in addition to the fused-ring cycloalkane, *trans*-decalin (YSI ∼ 105.5), studied previously.^49^

OHI showed benzene formation three times higher than that of *p*-menthane and DMCO, suggesting that benzene is likely formed directly from the fuel rather than through the recombination of intermediates. This is further supported by the relatively lower concentration of benzene precursors (Figure 9b–d). *p*-Menthane and DMCO exhibited comparable concentrations of benzene precursors and aromatics, with *p*-menthane systematically having slightly higher concentrations and no allene detected for DMCO. However, the intermediate pool of DMCO contained a greater variety of detected aromatics, albeit at low concentrations (Figure 7). *trans*-Decalin shows a trend similar to OHI, with higher concentrations of aromatics (benzene and toluene) and lower concentrations of benzene precursors (1,3-butadiene and propyne) compared to *p*-menthane and DMCO. This suggests that the direct formation of benzene may be generalized to all fused-ring cycloalkanes but appears to be a less favorable pathway for single-ring cycloalkanes. This is also consistent with the calculated IHDs of 2 for OHI and decalin and 1 for *p*-menthane and DMCO, where a high IHD results in higher benzene/toluene concentrations.^20^

**Figure 9:**
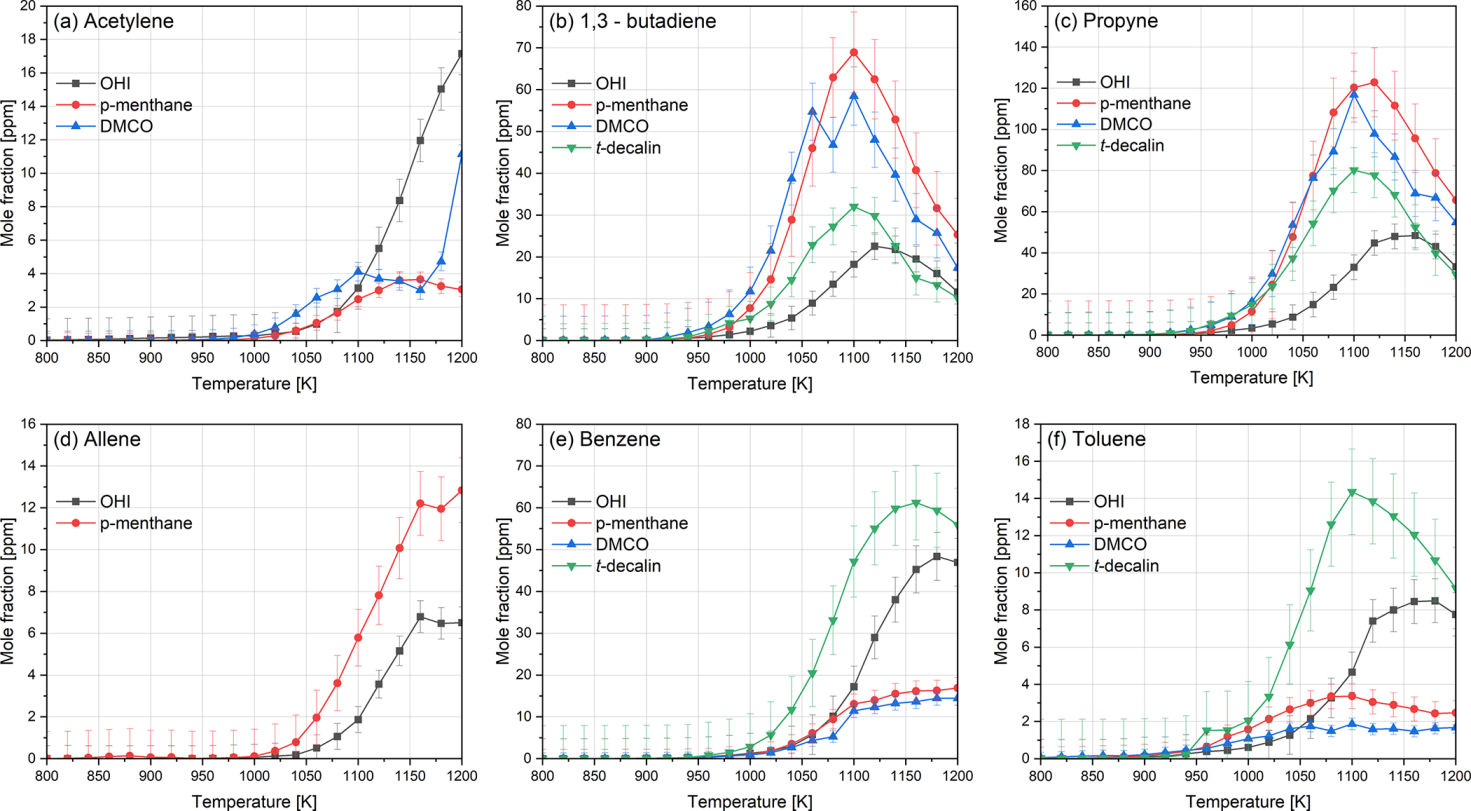
Mole fractions of soot-related intermediates; (a) acetylene, (b) 1,3-butadiene, (c) propyne, (d) allene, (e) benzene, and (f) toluene, obtained at 10 bar and phi = 3. *trans*-Decalin data are obtained elsewhere.^49^

For liquid fuel mixtures, the YSI of the mixture is observed to be a linear mole fraction weighted sum of the YSI of the individual components constituting the mixture.^58^ A similar approach was extended to gas-phase components to compare the YSIs of the flow reactor effluent mixture for a better understanding of the contribution to soot formation. Note that YSIs are measured in methane-doped coflow diffusion flames, where flame temperatures and equivalence ratios are high. The flow reactor experiments were performed under moderate conditions compared to those observed in the flame. The linear mole fraction weighted YSI of the effluent (YSI_eff_) was calculated with measured or estimated YSI values of the species identified in the effluent. The measured YSIs were obtained from,^46^ and estimates were obtained from the YSI prediction tool.^19,26^

The variation of the YSI_eff_ at various reactor temperatures is shown in Figure 10. YSI_eff_ reduces with temperature, as the parent molecule undergoes oxidation to various species. Many of these species, such as methane, ethane, CO, and CO_2_, have low YSI values. At high temperatures, the high concentrations of these species result in dilute mixtures, lowering the overall YSI of the mixture. Similar variations for different cycloalkanes correlate well with the parent molecule YSI. As the temperature increases, the contribution to YSI (i.e., percent of the species contributing to YSI_eff_) is enhanced for aromatics and other species exhibiting higher YSI values due to the increasing concentrations. The contributions of high-YSI species benzene and toluene at various temperatures are shown in Figure 11. At 1150 K and higher in Figure 10, the impact of increasing benzene concentrations for the OHI is evident. As mentioned above, the flow reactor can be operated only up to 1200 K, but if it were able to run at higher temperatures, the contribution of these species would likely be higher in accordance with established combustion/soot kinetics. Among the three cycloalkanes, the contribution of benzene to the YSI of OHI is higher, as explained above.

**Figure 10:**
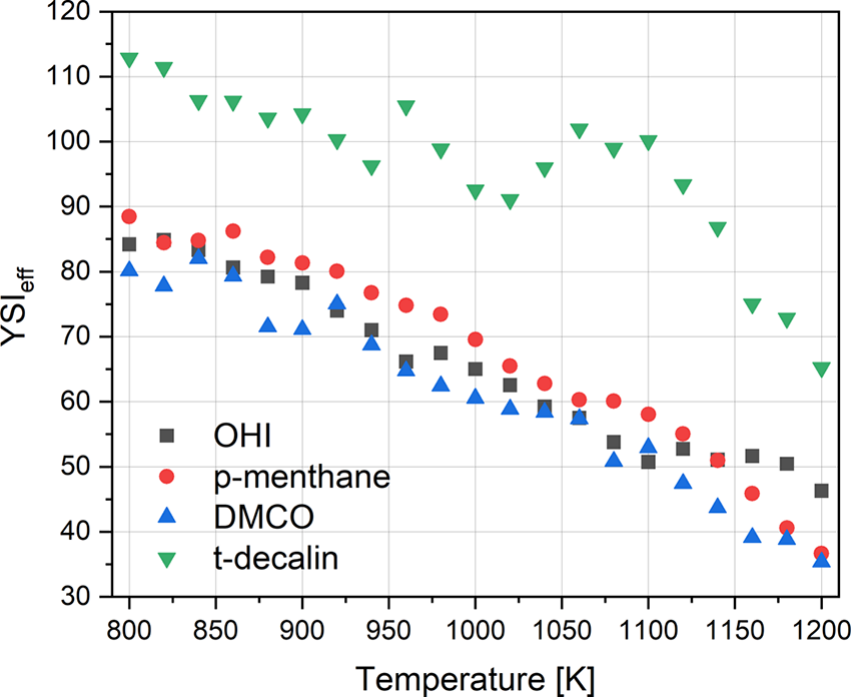
Linear mole fraction weighted YSI of the effluent for different cycloalkanes. *trans*-Decalin data are obtained elsewhere.^49^

**Figure 11:**
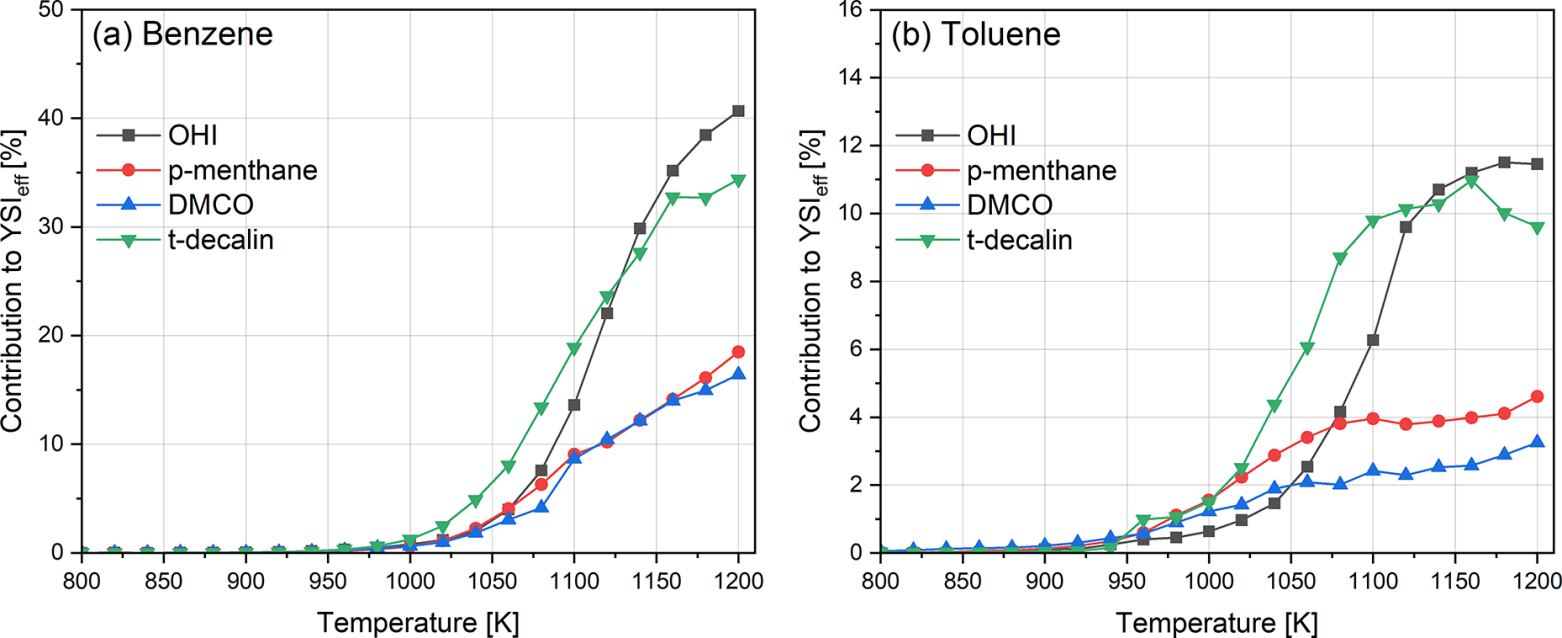
Contribution of high-YSI species, (a) benzene and (b) toluene, to YSI_eff_. *trans*-Decalin data are obtained elsewhere.^49^

## 4 Conclusions

This work investigated the combustion characteristics of promising cycloalkanes for jet fuels under soot precursor conditions with a pressurized laminar flow reactor. OHI, *p*-menthane, and DMCO were selected as representative cycloalkanes with distinct structural features. The flow reactor experiments were conducted at 10 bar under fuel-rich conditions to investigate the sooting behavior. The major detected intermediates and predominant reaction pathways were analyzed, focusing on limited fuel decomposition reactions at high temperatures, where most intermediates peaked. Key intermediates associated with benzene formation were compared across the three cycloalkanes to understand the measured YSI and contribution to the YSI of the effluent. OHI, similar to *trans*-decalin, exhibited the highest aromatic yields (benzene and toluene), while *p*-menthane and DMCO showed higher concentrations of ring-opening products such as 1,3-butadiene and propyne. The higher YSIs of the fused-ring cycloalkanes, *trans*-decalin (∼105.5) and OHI (94.2), suggest that stepwise dehydrogenation may play a greater role in aromatic formation for these compounds—a pathway that is indirect or absent in DMCO, leading to a lower YSI (85.0). The data presented in this study span a wide range of cycloalkane structures and are valuable for developing kinetic models to improve our understanding of cycloalkane combustion chemistry and sooting behavior.

## Supplementary Material









## Notes

Schematic of laminar flow reactor, flow rates of oxidation experiments, uncertainty quantification and detected species profiles at 10 bar and phi = 1. Schematics and more details on YSI measurements apparatus (PDF)
Excel sheets on flow reactor temperature profiles (XLSX)
Carbon balance and experimental data at phi = 3.0 (XLSX)
Experimental data at phi = 1.0 (XLSX)
