# Supplementary material for: Flow Reactor Study of the Soot Precursors of Novel Cycloalkanes as Synthetic Jet Fuel Compounds: Octahydroindene, p‑Menthane, and 1,4-Dimethylcyclooctane
Source: Energy Fuels. 2025 Oct 30;39(45):22048–60. doi: 10.1021/acs.energyfuels.5c03795 (PMC12621198; doi:10.1021/acs.energyfuels.5c03795)
Supplement: Supplementary file 1 [file ef5c03795_si_001.pdf]

Supporting Information to

Flow Reactor Study of the Soot Precursors of Novel Cycloalkanes as Synthetic Jet Fuel Compounds: Octahydroindene, p-Menthane, and 1,4-Dimethylcyclooctane

Samah Y. Mohamed†, Nimal Naser†, Zhanhong Xiang‡, Gina M. Fioroni†, Charles McEnally‡, Robert L. McCormick†

†National Renewable Energy Laboratory, Golden, CO 80401 United States

‡Department of Chemical and Environmental Engineering, Yale University

Figure S 1: Schematic of the NREL laminar flow reactor 2
Figure S 2: detected species in OHI oxidation at 10 bar and phi=1.0 4
Figure S 3: detected species in p-menthane oxidation at 10 bar and phi=1.0....5
Figure S 4: detected species in DMCO oxidation at 10 bar and phi=1.0....6
Figure S 5: Schematic Diagram of the YSI Apparatus 7
Figure S 6: Schematic Diagram of the YSI Burner 8
Figure S 7: Equilibration of the YSI Test Fuels with the Fuel Delivery System 10
Figure S 8: Linearity of YSI data....11

Table S 1: Flow rates of OHI oxidation runs....2
Table S 2: Flow rates of p-menthane oxidation runs 3
Table S 3: Flow rates of DMCO oxidation runs 3
Table S-4: Uncertainty values used in uncertainty quantification 4
Table S 5: Test Fuel Flowrates....9

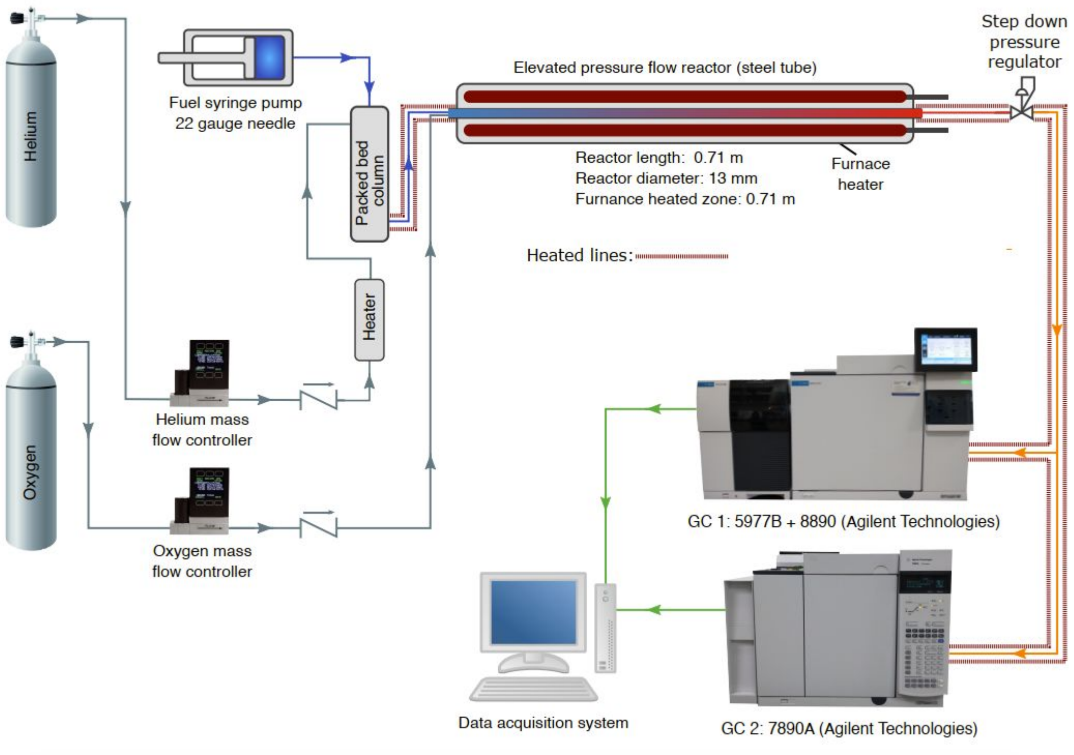

Figure S 1: Schematic of the NREL laminar flow reactor

Table S 1: Flow rates of OHI oxidation runs

T [K]	Fuel flowrate [ml/h]	Oxidizer flowrate [sccm]	Diluent flow rate [slpm]
800	3.637	41.536	41.808
820	3.548	40.523	40.789
840	3.463	39.558	39.817
860	3.383	38.638	38.891
880	3.306	37.760	38.007
900	3.233	36.921	37.163
920	3.162	36.118	36.355
940	3.095	35.350	35.581
960	3.030	34.613	34.840
980	2.969	33.907	34.129
1000	2.909	33.229	33.447
1020	2.852	32.577	32.791
1040	2.797	31.951	32.160
1060	2.745	31.348	31.553
1080	2.694	30.767	30.969
1100	2.645	30.208	30.406

Table S 2: Flow rates of p-menthane oxidation runs

T [K]	Fuel flowrate [ml/h]	Oxidizer flowrate [sccm]	Diluent flow rate [slpm]
800	4.481	47.927	41.802
820	4.372	46.758	40.782
840	4.268	45.644	39.811
860	4.169	44.583	38.885
880	4.074	43.570	38.002
900	3.984	42.602	37.157
920	3.897	41.675	36.349
940	3.814	40.789	35.576
960	3.735	39.939	34.835
980	3.658	39.124	34.124
1000	3.585	38.341	33.441
1020	3.515	37.590	32.786
1040	3.447	36.867	32.155
1060	3.382	36.171	31.549

Table S 3: Flow rates of DMCO oxidation runs

T [K]	Fuel flowrate [ml/h]	Oxidizer flowrate [sccm]	Diluent flow rate [slpm]
800	4.356	47.927	41.802
820	4.250	46.758	40.782
840	4.149	45.644	39.811
860	4.052	44.583	38.885
880	3.960	43.570	38.002
900	3.872	42.602	37.157
920	3.788	41.675	36.349
940	3.707	40.789	35.576
960	3.630	39.939	34.835
980	3.556	39.124	34.124
1000	3.485	38.341	33.441
1020	3.417	37.590	32.786
1040	3.351	36.867	32.155
1060	3.288	36.171	31.549
1080	3.227	35.501	30.964
1100	3.168	34.856	30.401

Table S-4: Uncertainty values used in uncertainty quantification

Parameter/Property	Value
Reactor length [m]	±0.020
Reactor diameter [m]	±0.0020
Pressure [bar]	±0.1
Temperature uncertainty [K]	± 6
Fuel delivery uncertainty [uL/min]	1.0
Diluent flow uncertainty [SLPM]	0.1
Oxidizer flow uncertainty [SCCM]	1.0
Diluent density [kg/m^3]	0.01
Oxidizer density [kg/m^3]	0.01

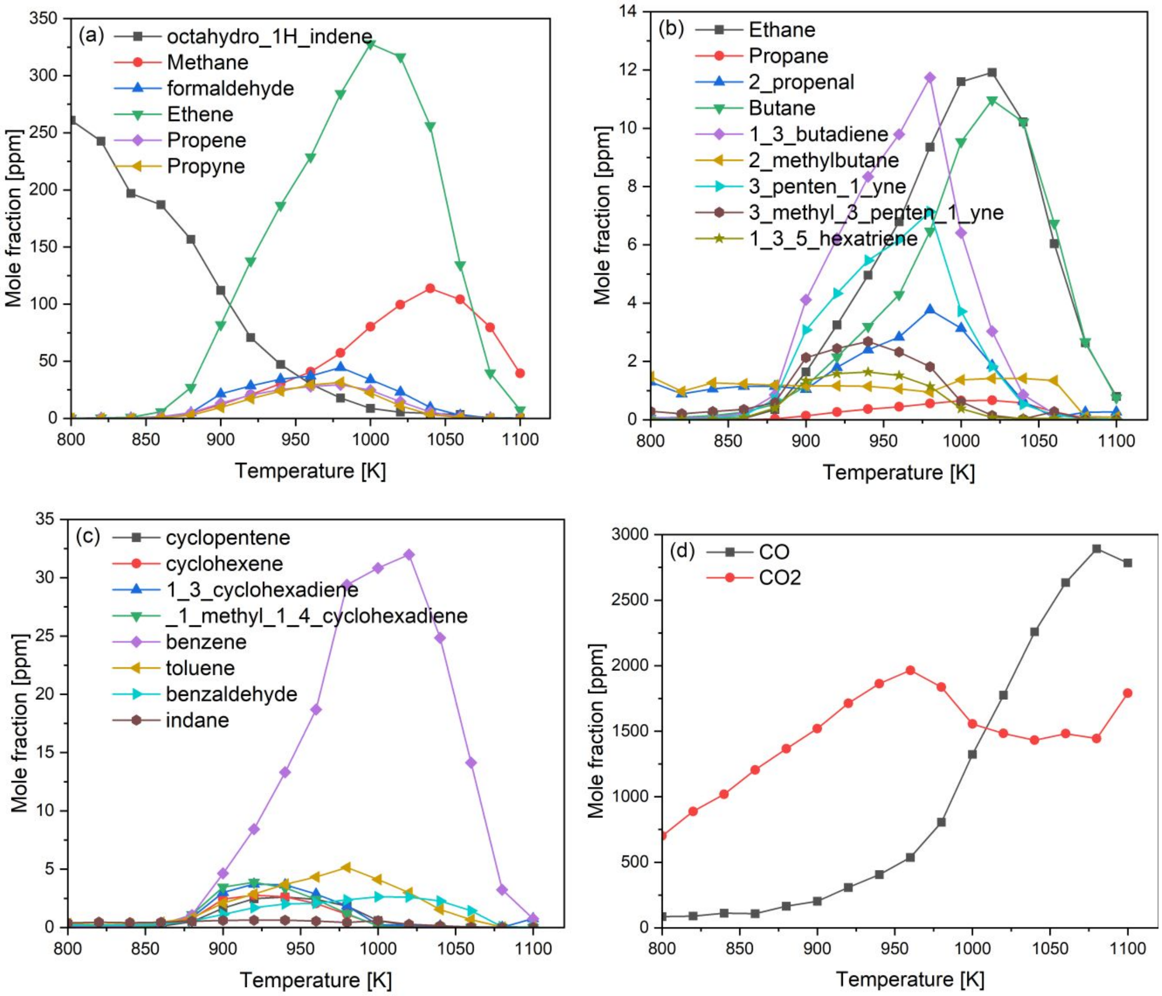

Figure S 2: detected species in OHI oxidation at 10 bar and phi=1.0

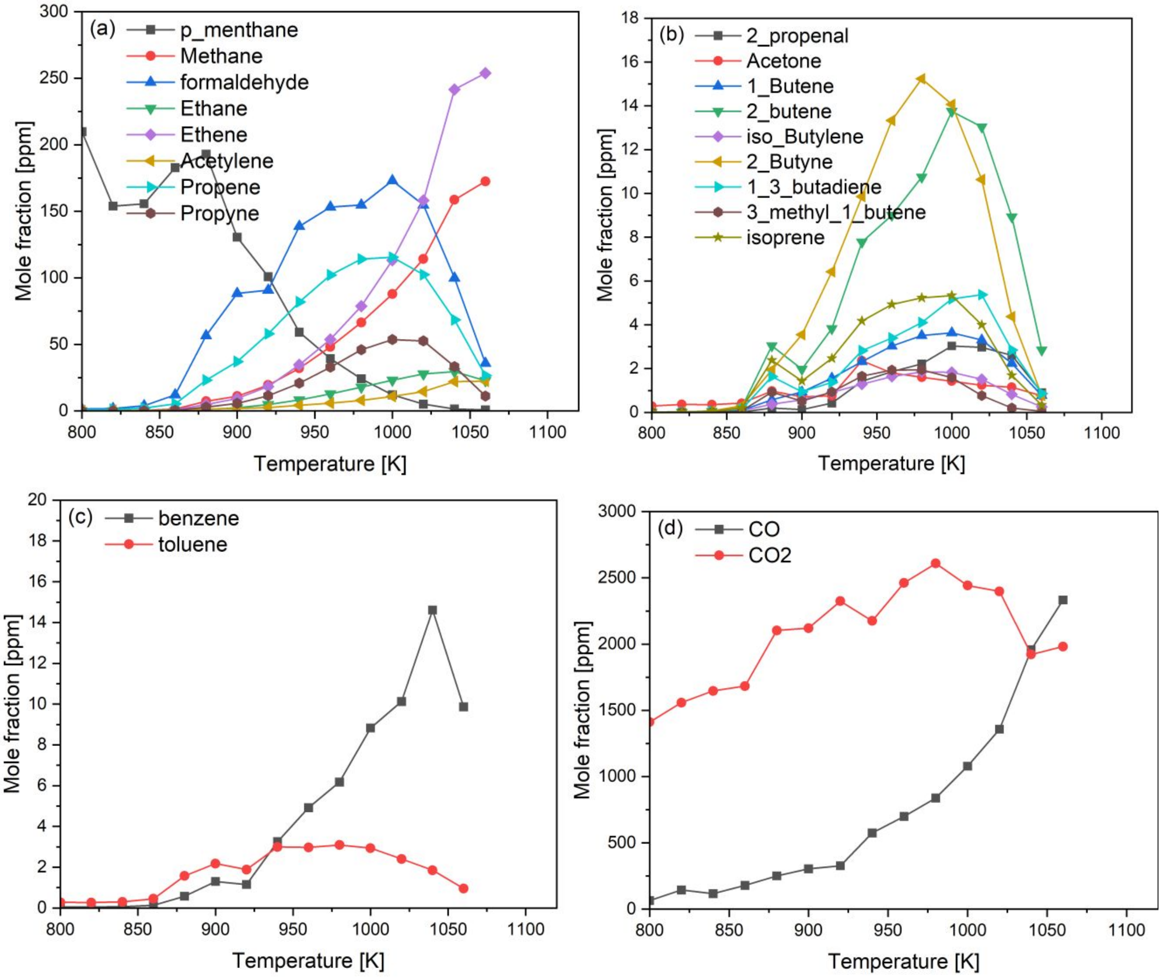

Figure S 3: detected species in p-menthane oxidation at 10 bar and phi=1.0

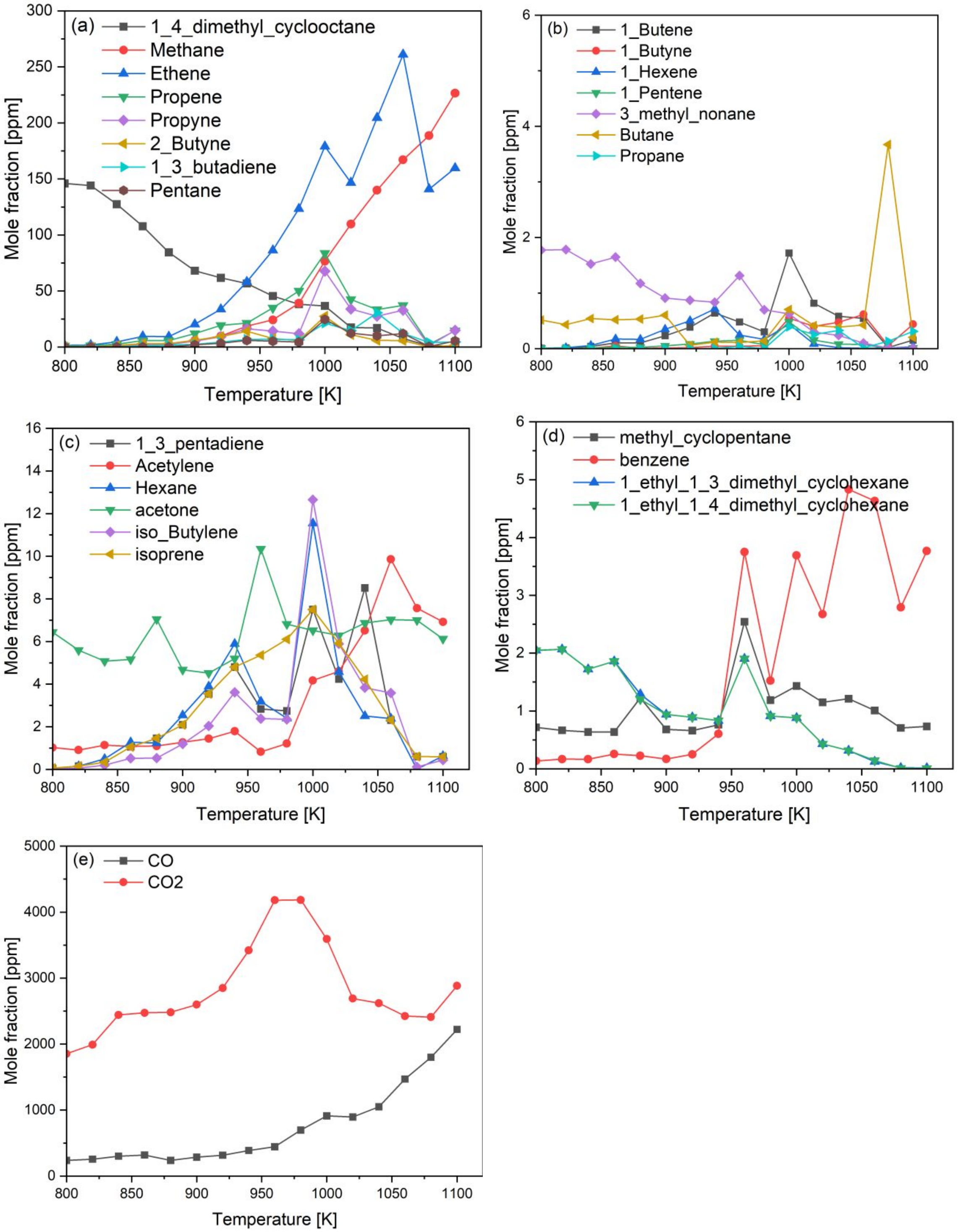

Figure S 4: detected species in DMCO oxidation at 10 bar and phi=1.0

Yield sooting index measurements

The figure below shows the apparatus used in the YSI measurements. Figure S 6 gives details of the specific burner. For all flames, the nominal gas-phase flowrate of the oxidizer (air) is 50,000 cm^3/min and the nominal gas-phase flowrates of the fuel components are 0.395 cm^3/min (test fuel), 282.425 cm^3/min (methane), and 112.18 cm^3/min (nitrogen). Mass flow controllers (MKS 1559A, Omega FMA5514ST) actively governed the flowrates of the gas-phase components. The controllers for CH_4 and N_2 were directly calibrated for the process gases with a 1000 cm^3 bubblemeter. A syringe pump (KDS Scientific 100) controlled the injection rates of the liquid test fuels into the gaseous CH_4/N_2 mixture. Table S 5 lists the liquid-phase flowrates calculated for each test fuel to produce the target gas-phase flowrate, and the property values used in these calculations. Resistive tapes heated all parts of the fuel line to at least 100 ℃ and the burner to 170 ℃. Given this heating, the dopants vaporized rapidly upon injection and were swept to the burner by the other fuel components. A PID controller maintained the burner temperature to within ±1 ℃, which was crucial since the soot concentrations in the flame depend sensitively on the initial temperature of the reactants.

The top of the figure shows the apparatus for measuring line-of-sight spectral radiance (L): a UV silica window embedded in the chimney transmits light from the flame to the rest of the setup; a fused silica biconvex lens focuses the light onto a 1 mm diameter circular aperture; an interference filter (Thorlabs FB660-10, frequency width at half maximum = 10 nm, center wavelength = 660 ± 2 nm) and an infrared-blocking filter (Schott KG2) isolate the light at 660 nm; and a red-enhanced photomultiplier tube (PMT; Oriel 77348) detects the light. An A/D converter (LeCroy LT342, 1 MΩ input impedance) samples the PMT output at 5 Hz. Each sample is an average of 50,000 8-bit measures recorded 2 μs apart.

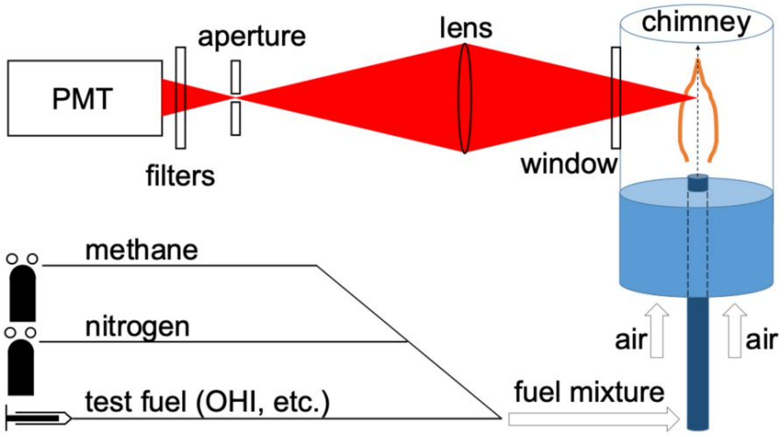

Figure S 5: Schematic Diagram of the YSI Apparatus

The figure below shows details of the burner used in the YSI measurements. All dimensions are given in the original units specified by the suppliers. Reference [1] provides a detailed burner description, including CAD drawings. This burner is being employed by multiple research groups as a standard configuration for experimental and numerical studies of soot formation under the auspices of the International Sooting Flame Workshop [2].

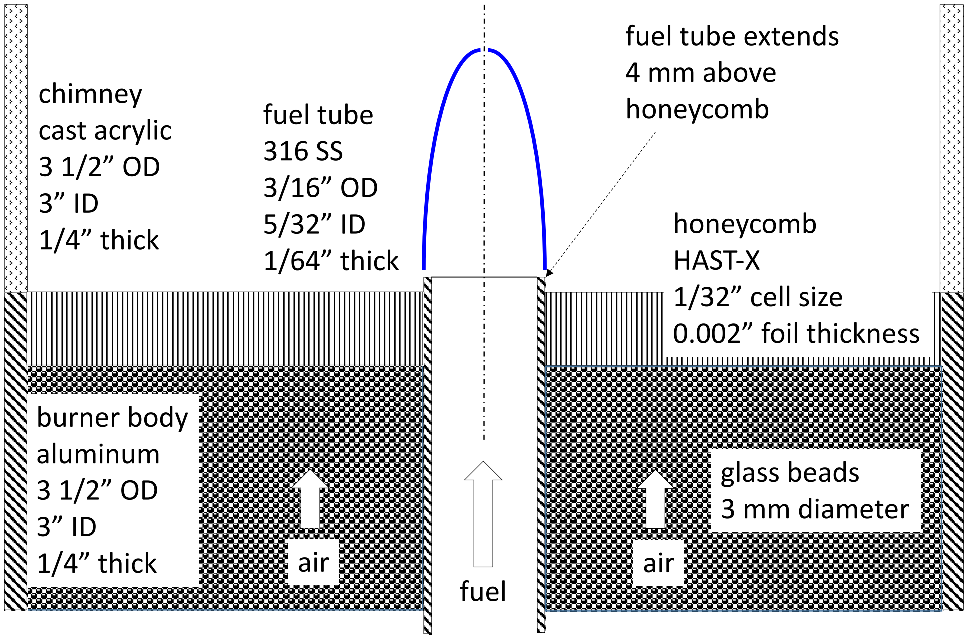

Figure S 6: Schematic Diagram of the YSI Burner

The table below lists the test fuel flowrates used in these measurements. The first column is the name of each test fuel or reference fuel. The second column is the relative molecular mass M_r for each test fuel. The third column is the liquid-phase mass density ρ for each test fuel. The values of ρ for T, H, and ISO are averages of the values from the vendor and [3-5]. These values were measured at a range of temperatures from 20 °C to 25 °C. The values for ρ for all other test fuels were estimated with the group contribution method in [6]. The fourth column is the liquid phase volumetric flowrate Q for each test fuel. These values were input to the syringe pump during the measurements. They were calculated to give a vapor phase volumetric flowrate of 0.395 cm^3/min at 296.4 K and 101.7 kPa—typical ambient conditions in our laboratory—using the ideal gas law and the values listed for M_r and ρ. The units and number of significant figures of Q match the required input to the syringe pump.

Table S 5: Test Fuel Flowrates

Test Fuel	M_r (g/mol)	ρ (g/mL)	Q (μL/h)
octahydroindene (OHI)	124.223	0.879	138.2
toluene (T)	92.138	0.869	103.7
Isooctane (ISO)	114.229	0.691	161.6
n-heptane (H)	100.202	0.686	142.9

The figure below addresses the adsorption of the test fuels onto the walls of the fuel lines and burner. The vertical axis is LSSR signal. The horizontal axis is time *t*, with *t* = 0 corresponding to starting the syringe pump. The blue line is the instantaneous signal measured for one trial of OHI. The red line is the steady-state signal, determined by averaging the instantaneous signal from t = 300 to 600 s. The instantaneous signal is initially lower than the steady-state value due to net adsorption of OHI onto the initially uncoated walls. However, the signal asymptotes to the steady-state value within about 130 s, which demonstrates that OHI achieves adsorption/desorption equilibrium with the walls on this timescale. The other test fuels have boiling points lower than OHI, so they also achieved equilibrium within 130 s. Once this equilibrium is achieved, there is no net loss of the test fuel to the walls, so the test fuel concentration entering the flame is the same as the concentration originally injected into the fuel mixture.

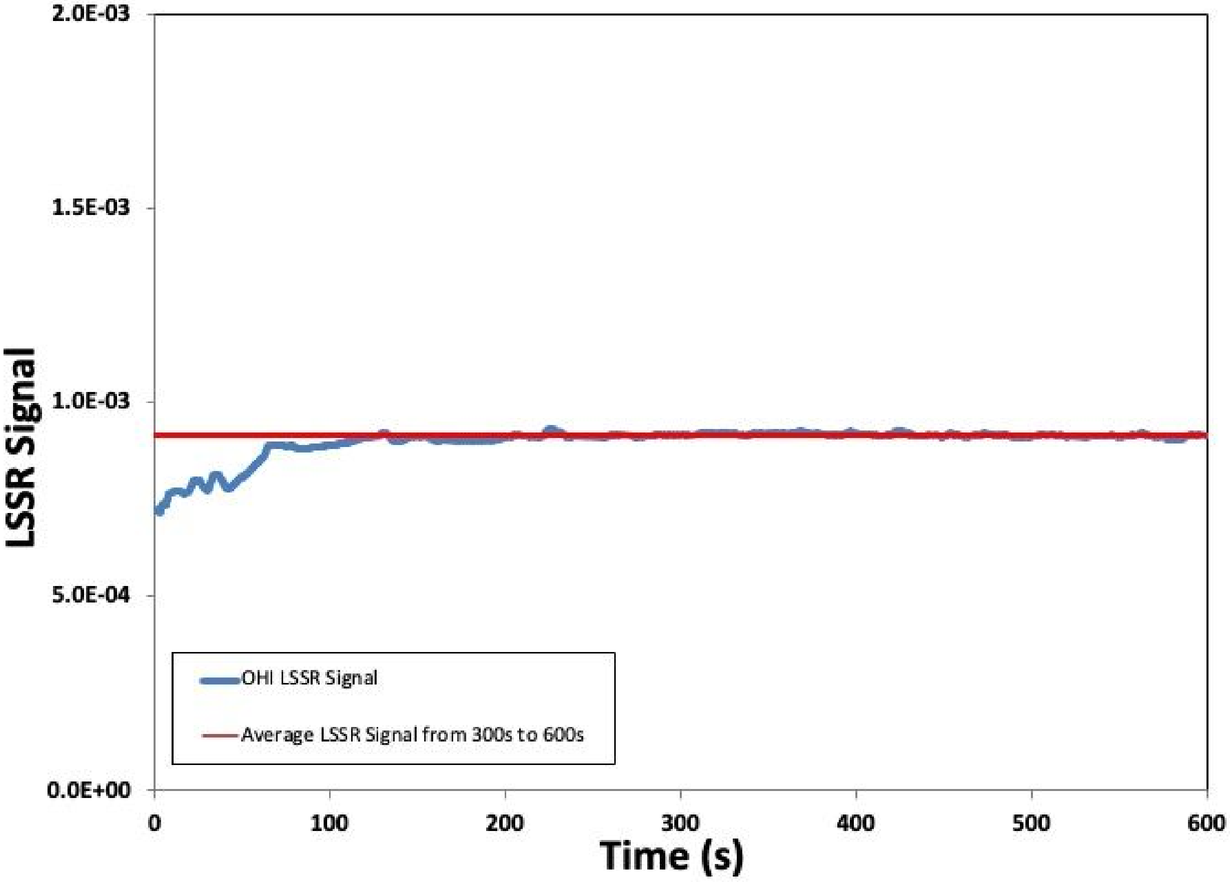

Figure S 7: Equilibration of the YSI Test Fuels with the Fuel Delivery System

The figure below shows the LSSR signal measured for a flame doped with decalin as a function of the dopant mole fraction. The data closely fits a linear trend (R^2 = 0.9987). This observation demonstrates that all the dopant was evaporating and reaching the flame—if the dopant had been condensing in the fuel lines, then the signal would have flattened out as the dopant concentration exceeded the saturation concentration. The other dopants including OHI have boiling points comparable to or lower than decalin, so they would not have condensed either.

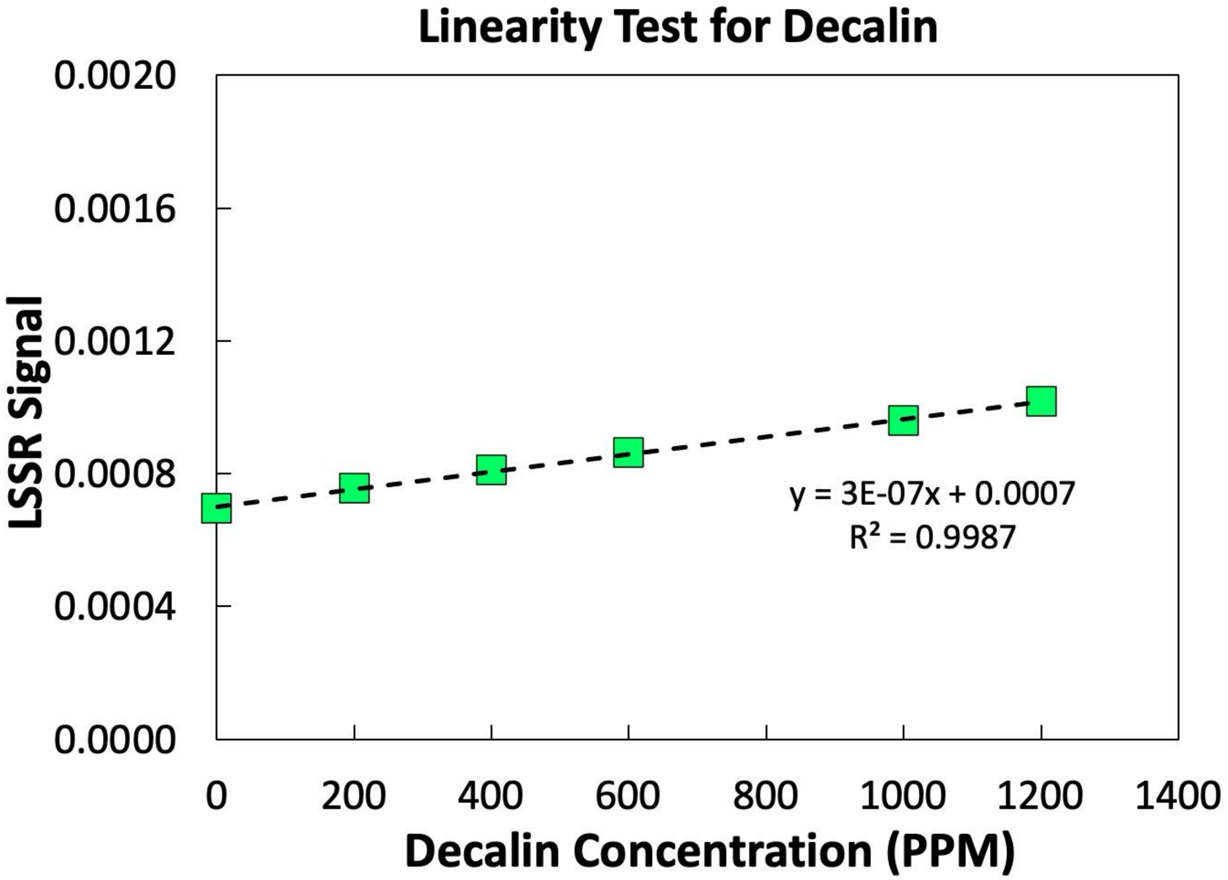

Figure S 8: Linearity of YSI data

References:

[1] J. Gau, D. Das, C. McEnally, D. Giassi, N. Kempema, M. Long, Yale Coflow Burner Information and CAD Drawings, (2017) DOI:10.6084/m9.figshare.5005007.v1.

[2] B. Franzelli, M. Roussillo, P. Scoflaire, J. Bonnety, R. Jalain, T. Dormieux, et al., Multi-diagnostic soot measurements in a laminar diffusion flame to assess the ISF database consistency, Proc. Combust. Inst. 37 (2019) 1355–1363.

[3] C.L. Yaws, Yaws' Handbook of Physical Properties for Hydrocarbons and Chemicals, (2008).

[4] J.R. Rumble, CRC Handbook of Chemistry and Physics, 101st Edition, (2019).

[5] DIPPR Project 801 - Full Version, Design Institute for Physical Property Research/AIChE, (2019).

[6] D. Mathieu, R. Bouteloup, Reliable and versatile model for the density of liquids based on additive volume increments, Ind. Eng. Chem. Res. 55 (2016) 12970–12980.
